# Supplementary material for: Herbicidal and Antibacterial Secondary Metabolites Isolated from the Nicotiana tabacum-Derived Endophytic Fungus Aspergillus japonicus TE-739D
Source: Plants (Basel). 2025 Jan 9;14(2):173. doi: 10.3390/plants14020173 (PMC11768264; doi:10.3390/plants14020173)
Supplement: Supplementary file 1 [file plants-14-00173-s001.zip › plants-3395033-supplementary.pdf]

Supporting Information

Herbicidal and Antibacterial Secondary Metabolites Isolated from the *Nicotiana tabacum*-Derived Endophytic Fungus *Aspergillus japonicus* TE-739D

Haisu Wang [1], Xiaolong Yuan [1], Xinrong Huang [1], Peng Zhang [1,*] and Gan Gu [1,*]

1 Tobacco Research Institute of Chinese Academy of Agricultural Sciences, Qingdao 266101, China; haisu0815@163.com (H. W.); yuanxiaolong@caas.cn (X. Y.); hxr1352460520@163.com (X. H.);

* Correspondence: zhangpeng@caas.cn (P. Z.); gugan@caas.cn (G. G.)

Contents

Figure S1. ^{1}H NMR spectrum of **1** (DMSO-d_6, 500 MHz) 3

Figure S2. ^{13}C NMR spectrum of **1** (DMSO-d_6, 125 MHz) 3

Figure S3. HSQC spectrum of **1** 4

Figure S4. ^{1}H-^{1}H COSY spectrum of **1** 4

Figure S5. HMBC spectrum of **1** 5

Figure S6. NOESY spectrum of **1** 5

Figure S7. UV spectrum of **1** 6

Figure S8. HRESIMS spectrum of **1** 6

Figure S9. ^{1}H NMR spectrum of **2** (CD_3OD, 500 MHz) 7

Figure S10. ^{13}C NMR spectrum of **2** (CD_3OD, 125 MHz) 7

Figure S11. HSQC spectrum of **2** 8

Figure S12. ^{1}H-^{1}H COSY spectrum of **2** 8

Figure S13. HMBC spectrum of **2** 9

Figure S14. NOESY spectrum of **2** 9

Figure S15. UV spectrum of **2** 10

Figure S16. HRESIMS spectrum of **2** 10

Figure S17. Linear regression between Exp. δ_C (x) and Calc. δ_C (y) of **2a1** and **2b1**.11

Figure S18. Inhibitory activity of **1** at different concentrations on seed germination of *Eleusine indica.* 11

Figure S19. Inhibitory activity of **2** at different concentrations on seed germination of *Amaranthus retroflexus* L. 11

Table S1. GIAO ^{13}C NMR calculation of **2a1** and **2b1** 12

Table S2. Inhibition rate of **1** on *Eleusine indica* seed germination 13

Table S3. Inhibition rate of **2** on *Amaranthus retroflexus* L. seed germination 13

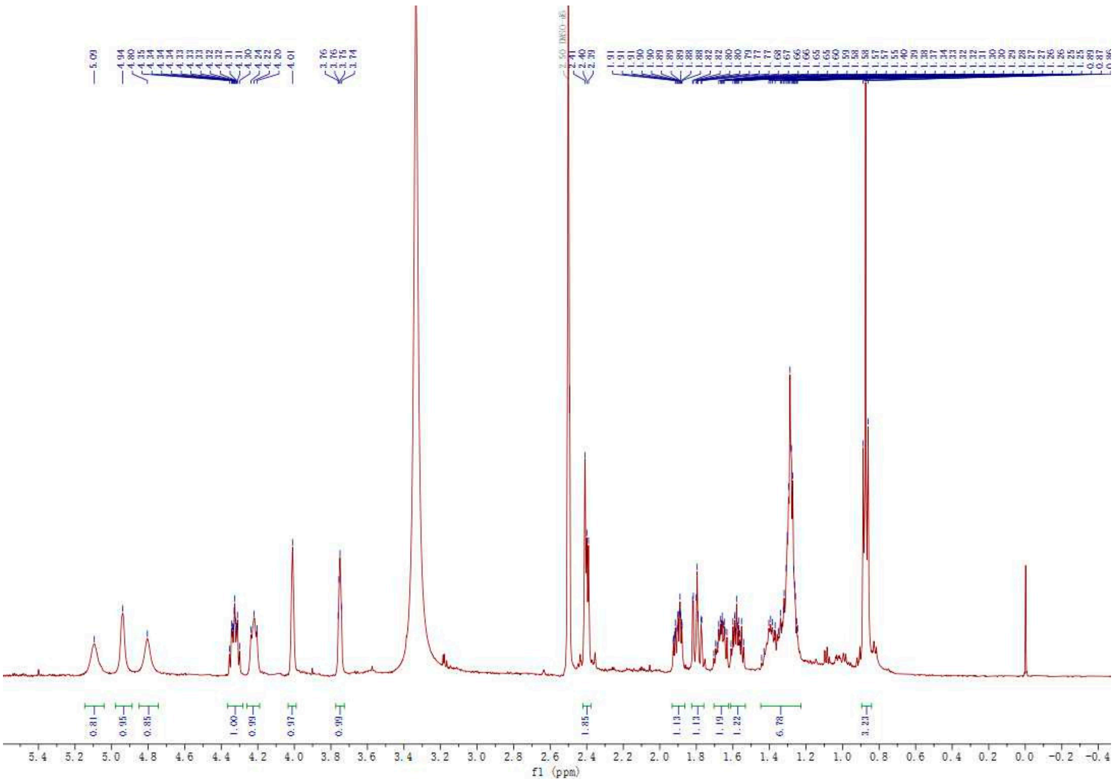

Figure S1. ^{1}H NMR spectrum of **1** (DMSO-d_6, 500 MHz)

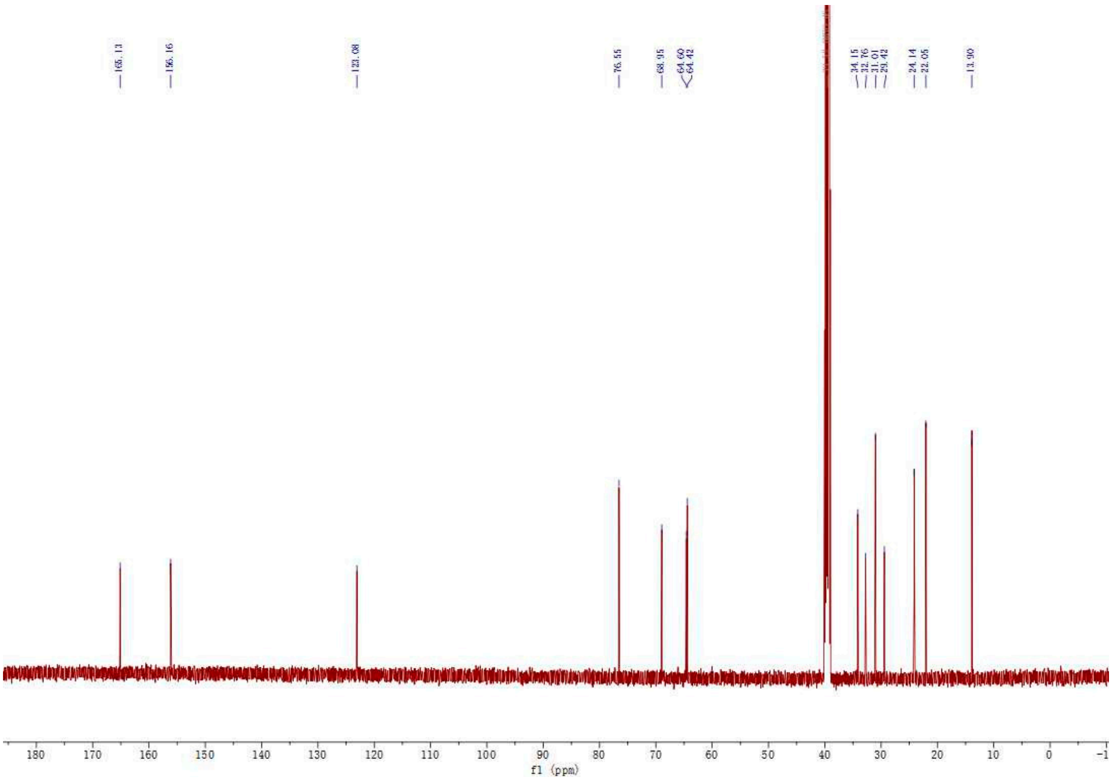

Figure S2. ^{13}C NMR spectrum of **1** (DMSO-d_6, 125 MHz)

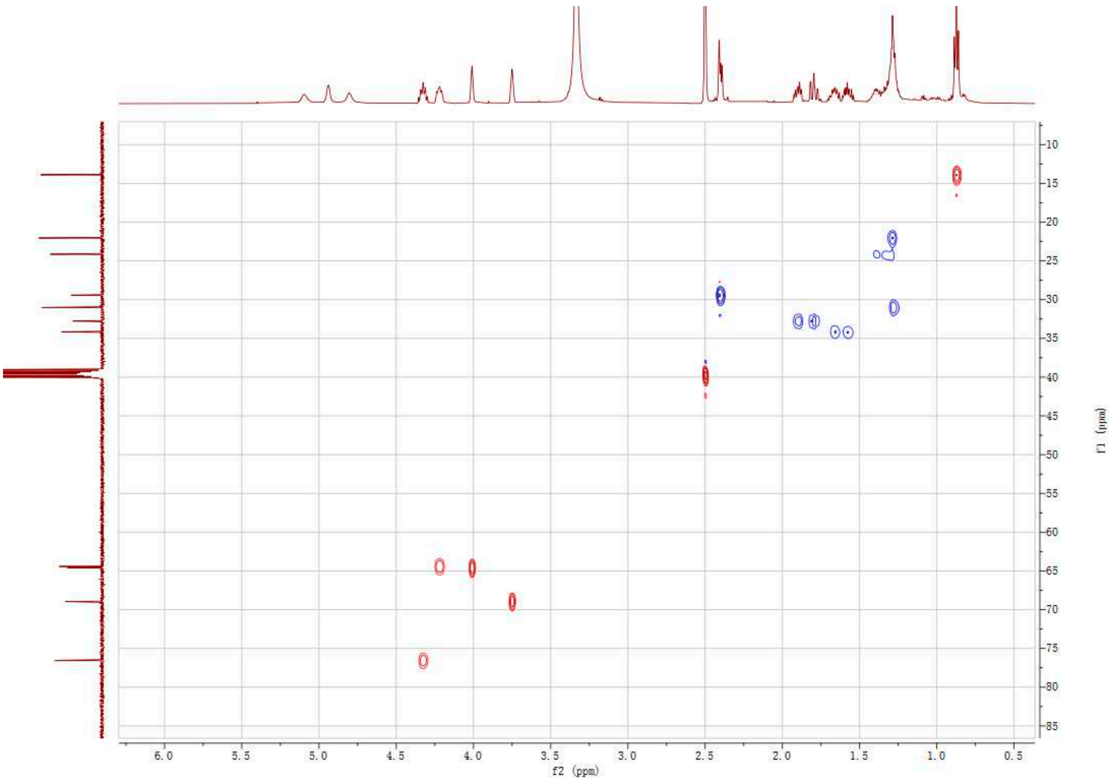

Figure S3. HSQC spectrum of **1**

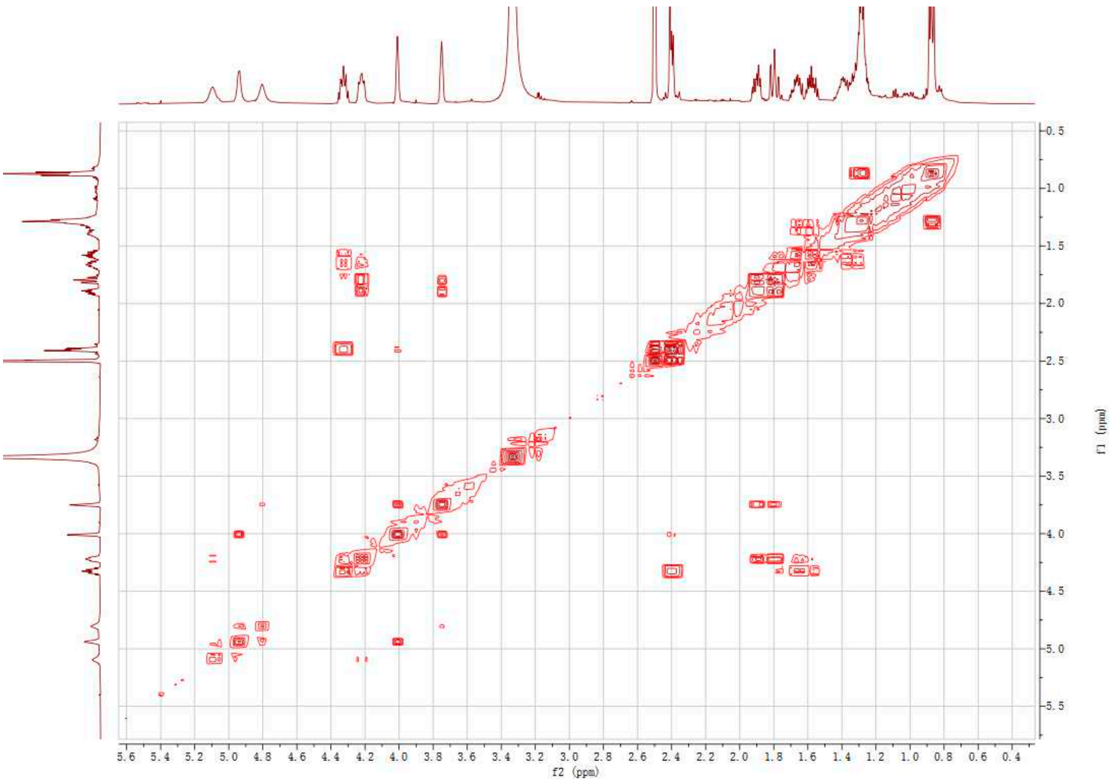

Figure S4. ^{1}H-^{1}H COSY spectrum of **1**

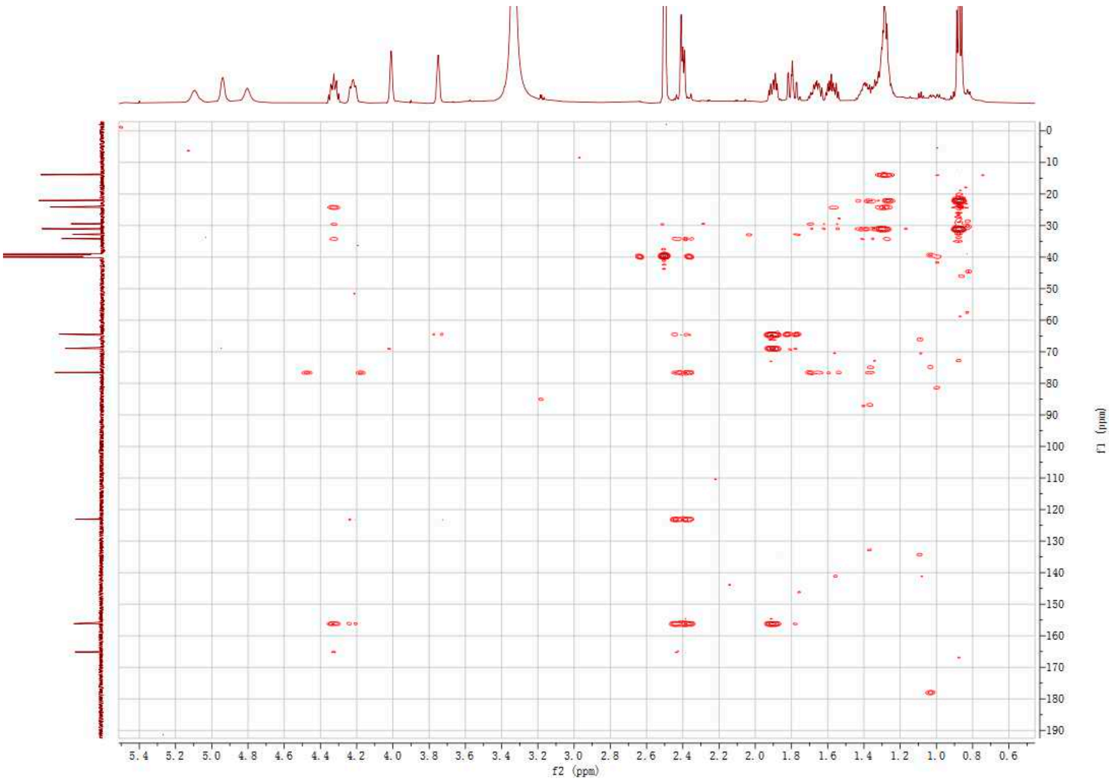

Figure S5. HMBC spectrum of **1**

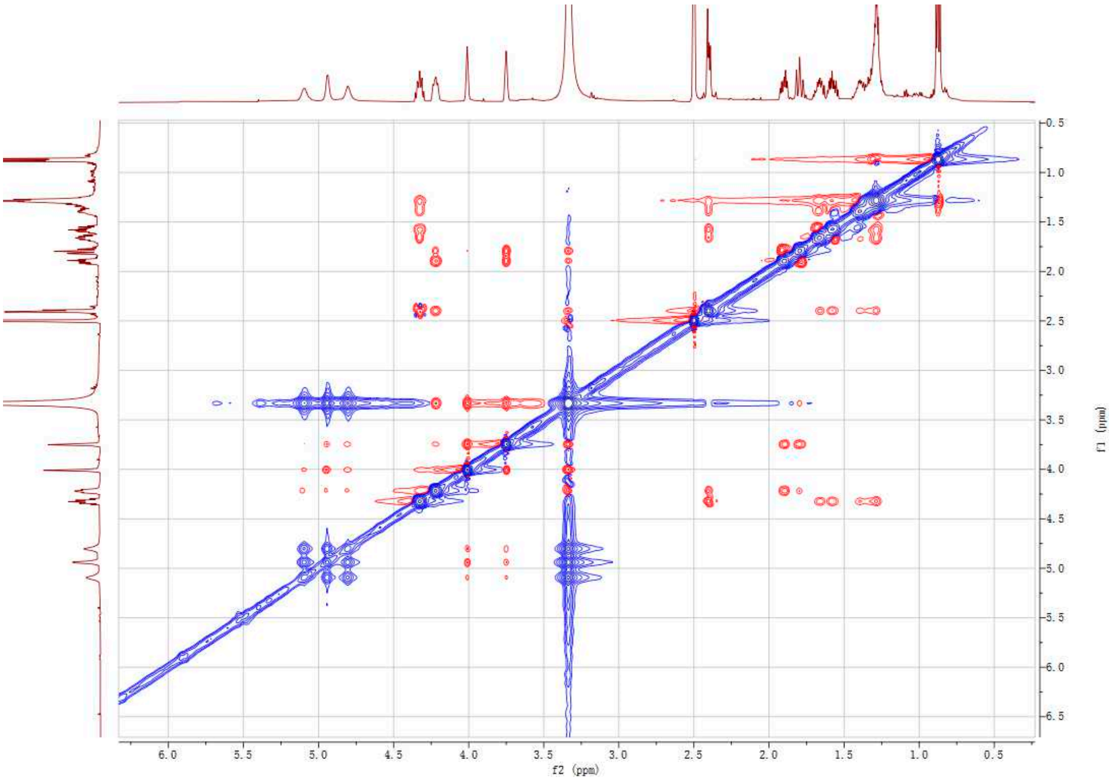

Figure S6. NOESY spectrum of **1**

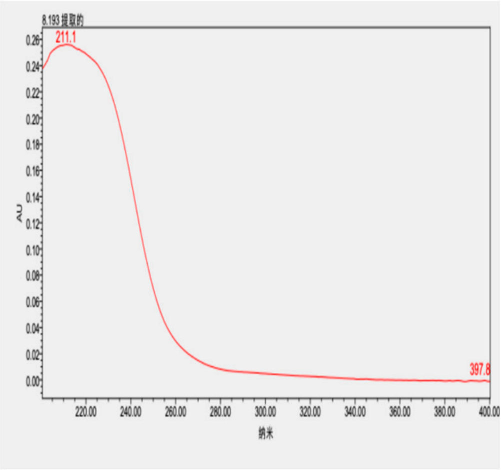

Figure S7. UV spectrum of **1** (100% MeOH/H_2O, extracted from HPLC-DAD data)

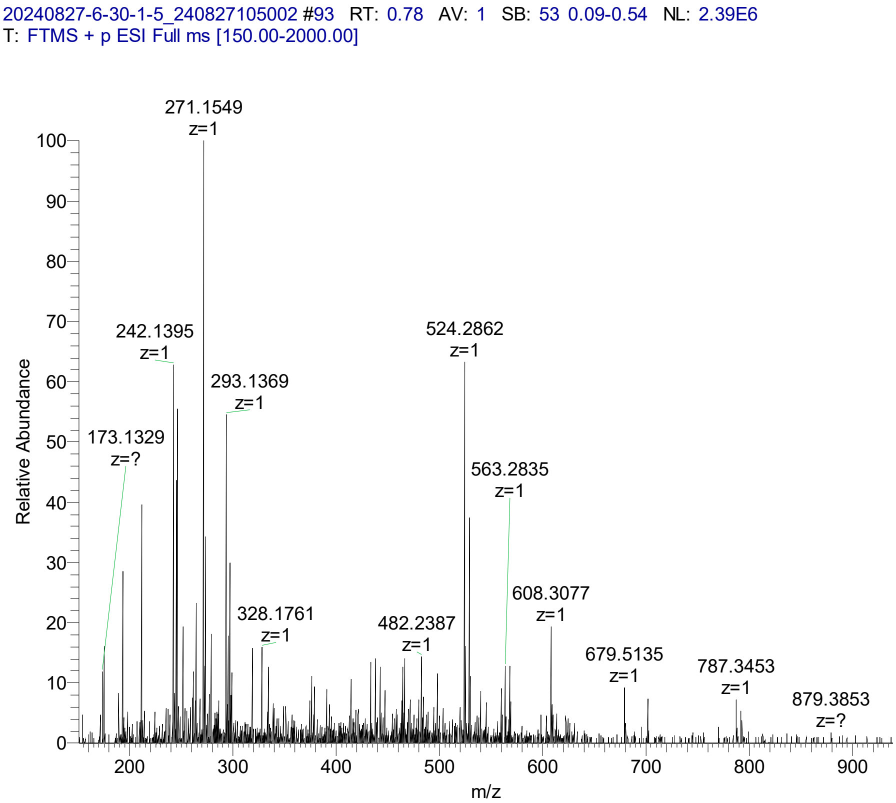

Figure S8. HRESIMS spectrum of **1**

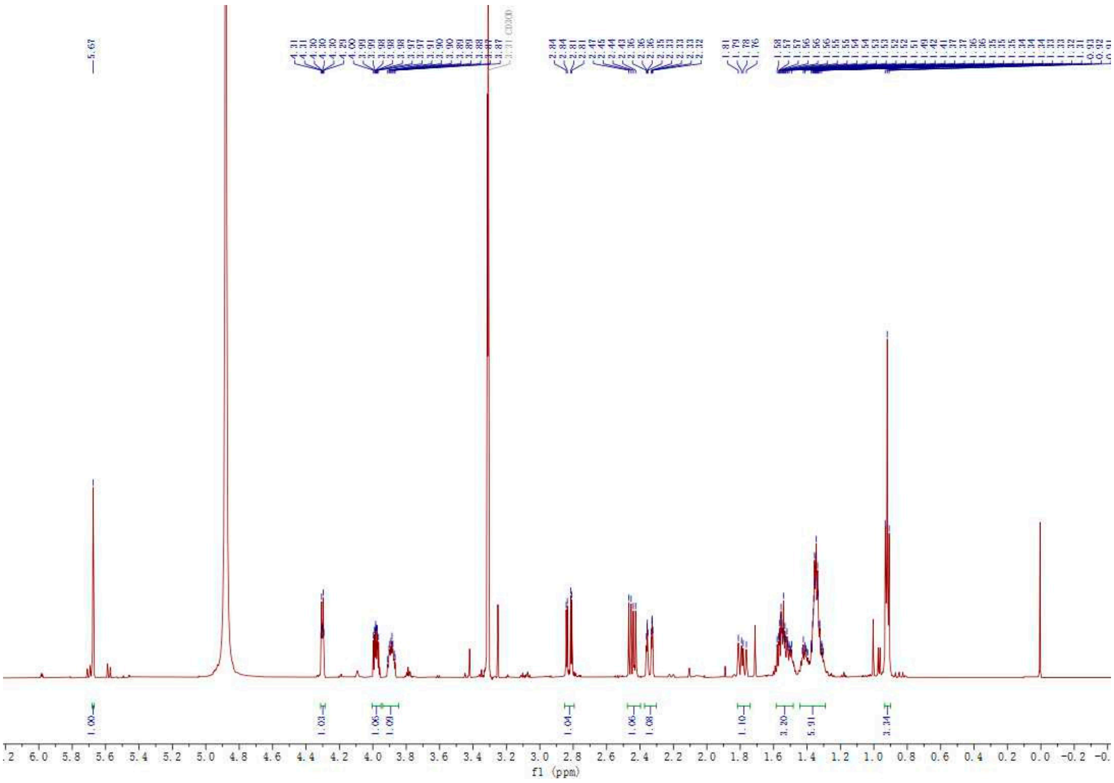

Figure S9. ^{1}H NMR spectrum of **2** (CD_3OD, 500 MHz)

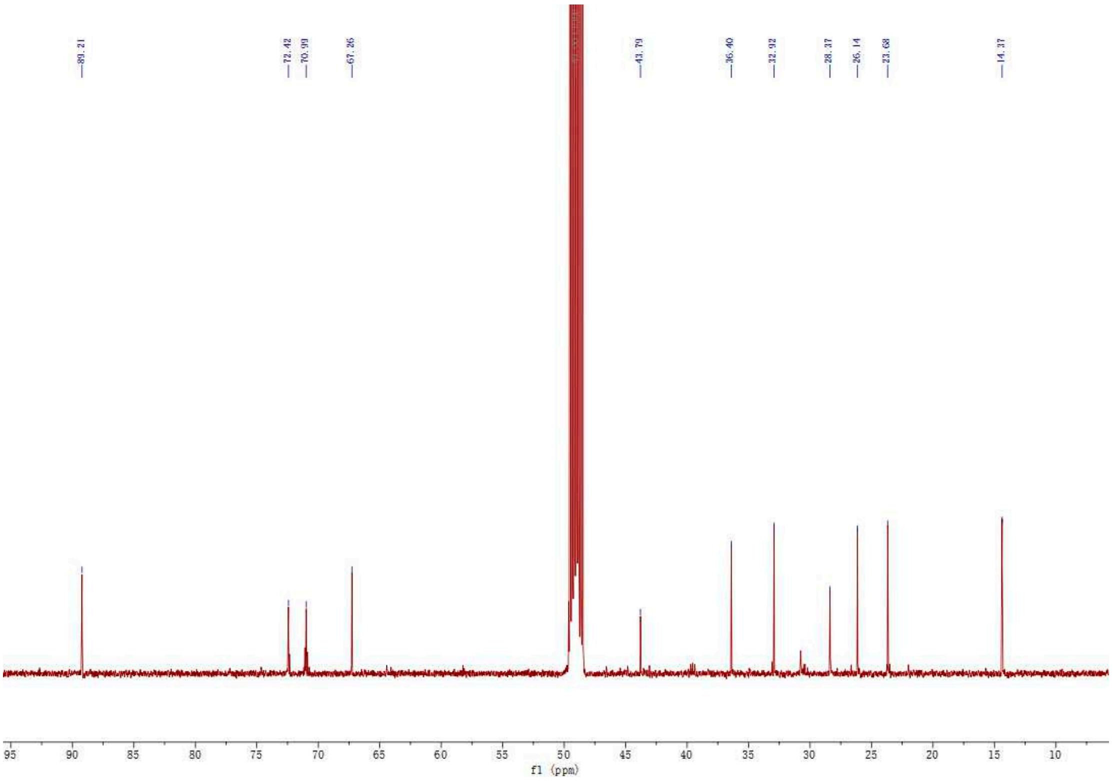

Figure S10. ^{13}C NMR spectrum of **2** (CD_3OD, 125 MHz)

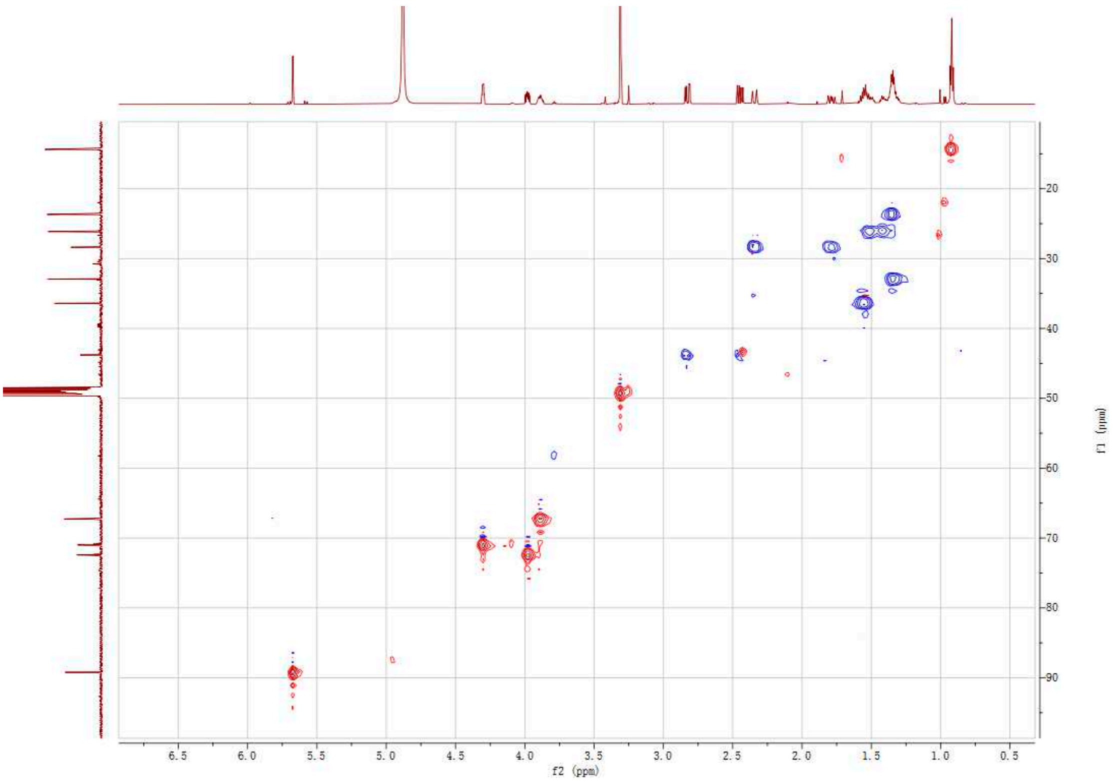

Figure S11. HSQC spectrum of **2**

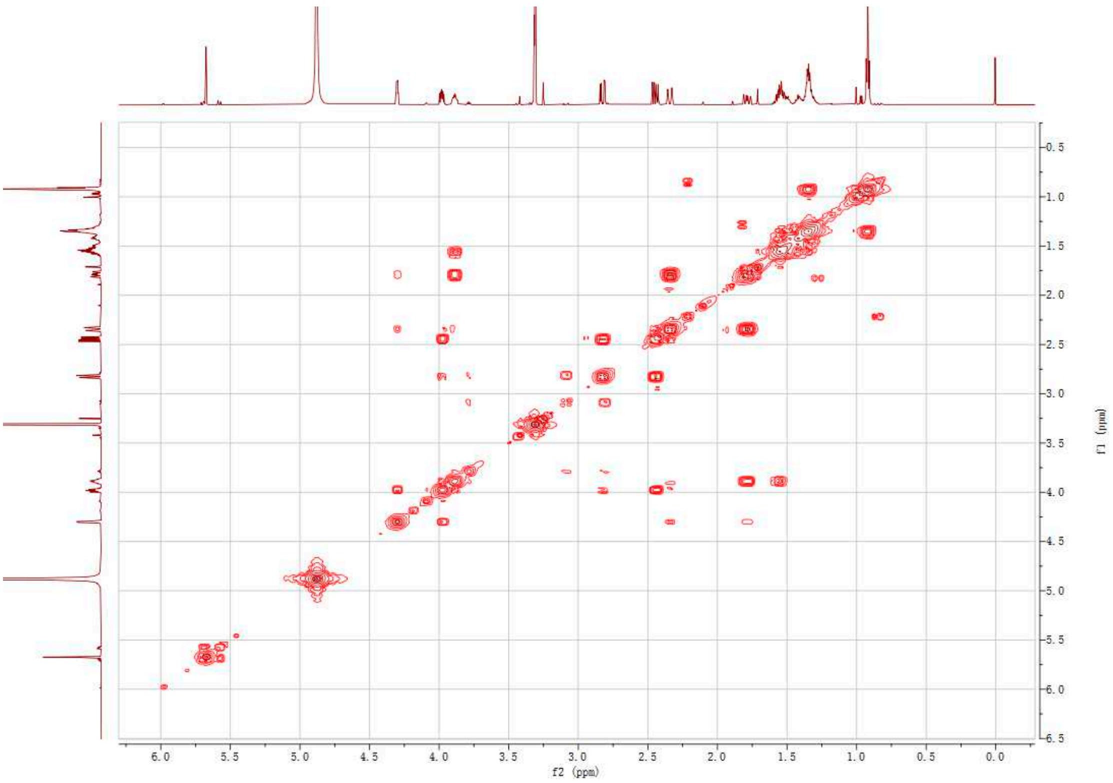

Figure S12. ^{1}H-^{1}H COSY spectrum of **2**

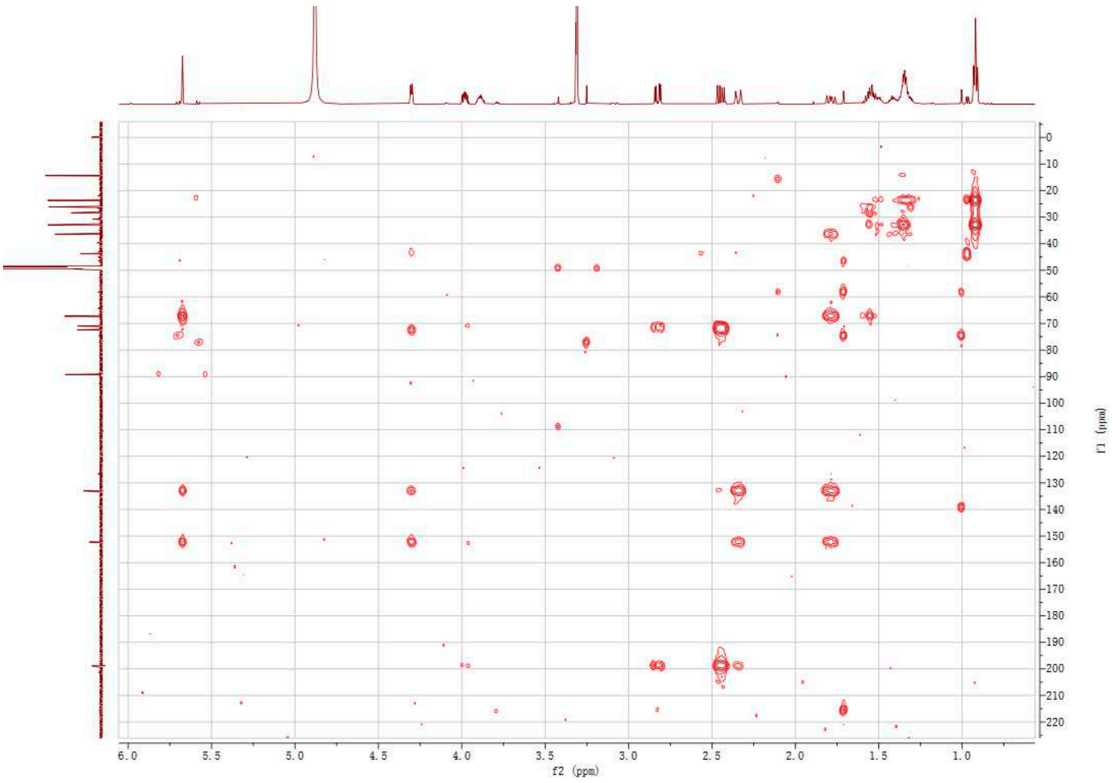

Figure S13. HMBC spectrum of **2**

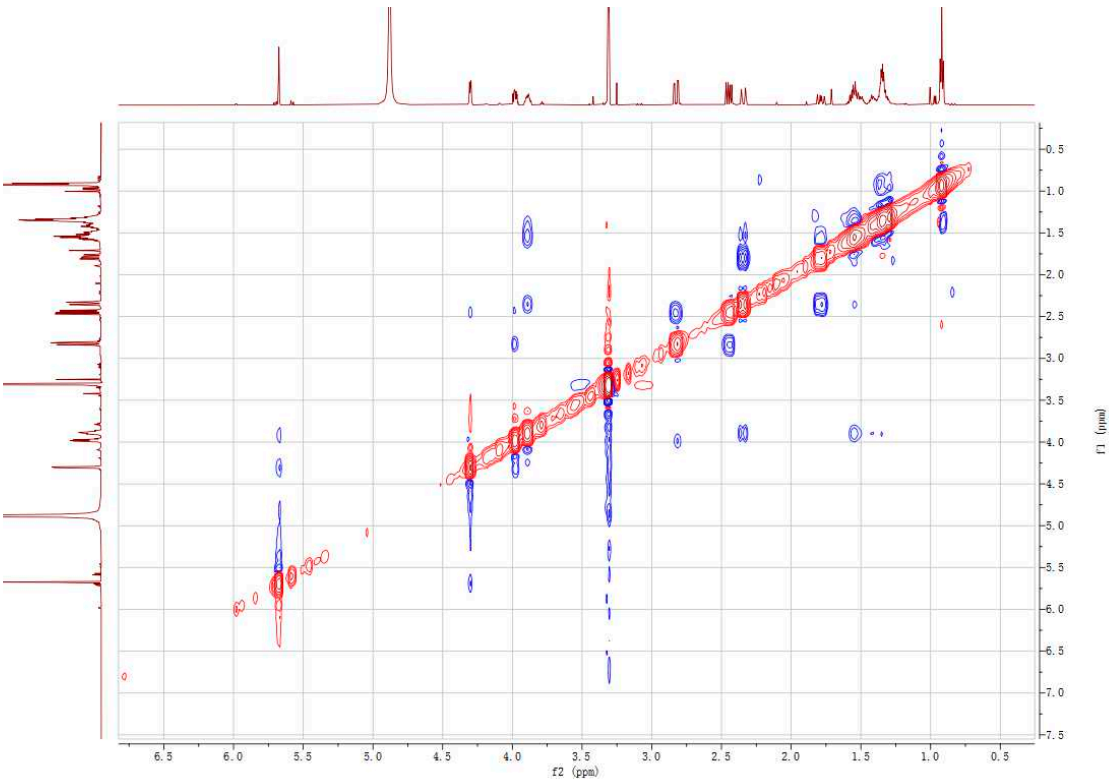

Figure S14. NOESY spectrum of **2**

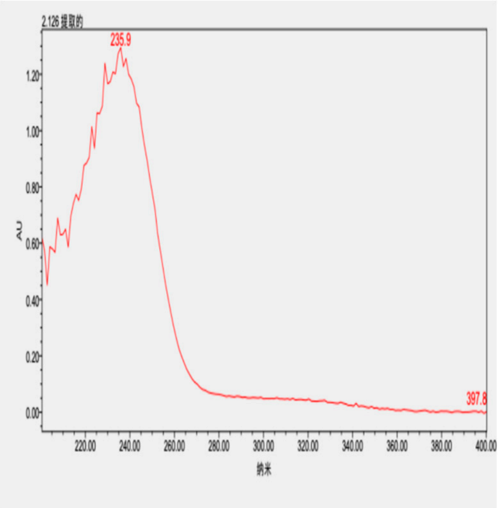

Figure S15. UV spectrum of **2** (100% MeOH/H_2O, extracted from HPLC-DAD data)

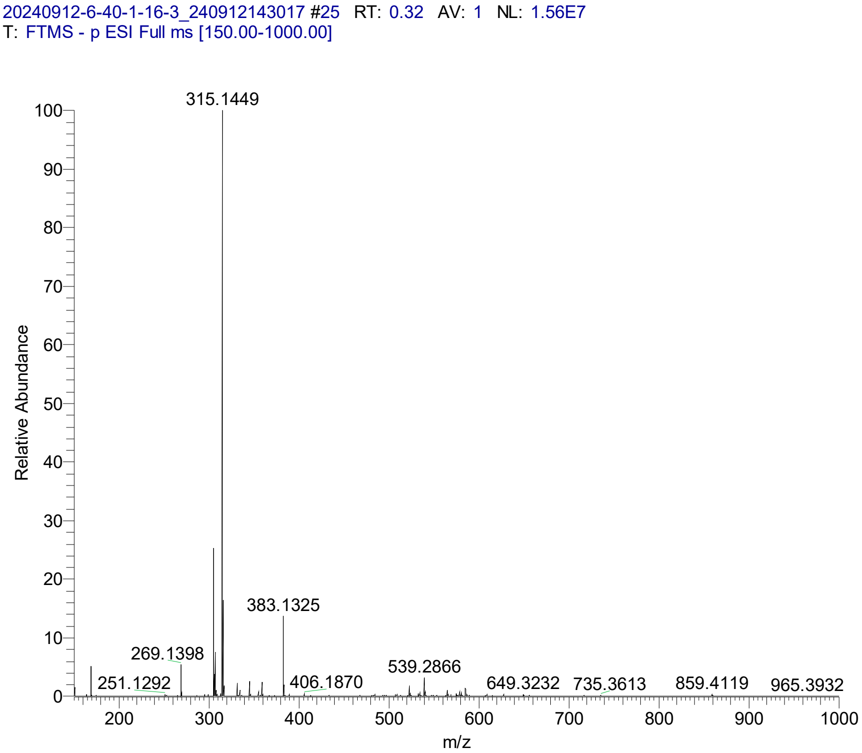

Figure S16. HRESIMS spectrum of **2**

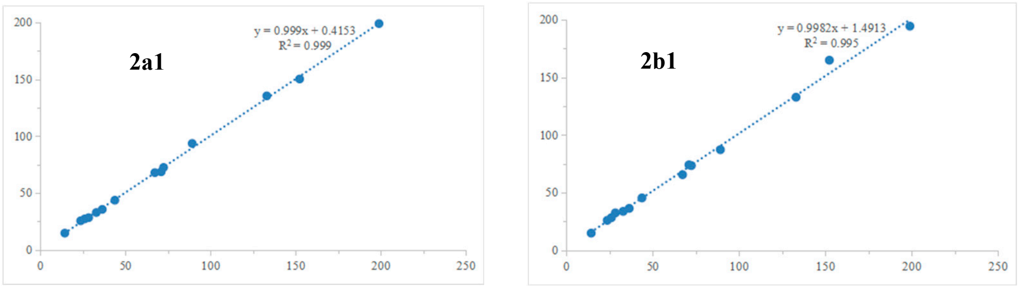

Figure S17. Linear regression between Exp. δ_C (x) and Calc. δ_C (y) of **2a1** and **2b1**.

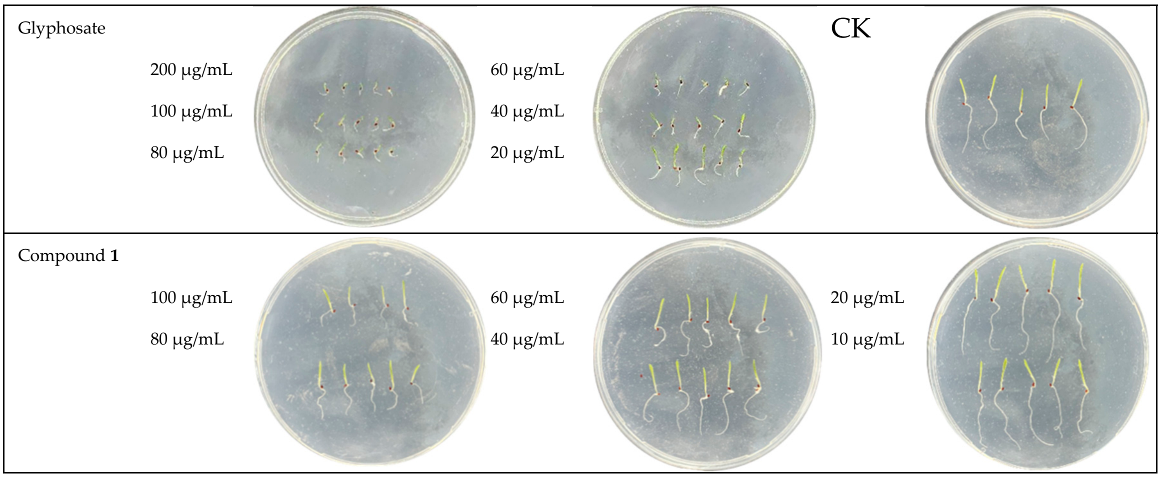

Figure S18. Inhibitory activity of **1** at different concentrations on seed germination of *Eleusine indica.*

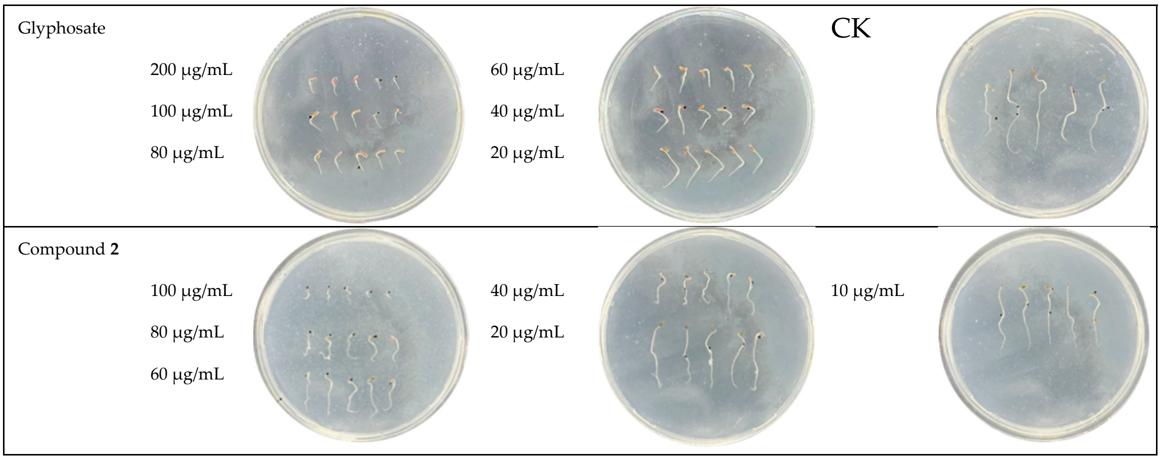

Figure S19. Inhibitory activity of **2** at different concentrations on seed germination of *Amaranthus retroflexus* L.

Table S1. GIAO ^{13}C NMR calculation of **2a1** and **2b1**

Position	Exp. δ_C	Cal. δ_C[a] (**2a1**)	$\Delta\delta$ (cal. − exp.)	Cal. δ_C[a] (**2b1**)	$\Delta\delta$ (cal. − exp.)
1	14.4	14.7	0.3	14.3	-0.1
2	23.7	25.5	1.8	25.7	2.0
3	32.9	32.8	-0.1	33.5	0.6
4	26.1	27.2	1.1	27.4	1.3
5	36.4	35.5	-0.9	37.0	0.6
6	67.3	67.9	0.6	72.8	5.5
7	28.4	28.3	-0.1	28.1	-0.3
8	133.0	135.4	2.4	134.4	1.4
9	198.9	199.0	0.1	199.3	0.4
10	43.8	43.6	-0.2	43.3	-0.5
11	72.4	72.4	0.0	72.4	0.0
12	71.0	68.6	-2.4	66.7	-4.3
13	152.3	150.2	-2.1	150.2	-2.1
14	89.2	93.6	4.4	95.3	6.1
		RMSD	1.4	RMSD	2.1
		MAE	0.8	MAE	1.1

[a] ^{13}C NMR calculations were performed at the mpw1pw91/6-311+G(2d,p) (PCM=MDSO)//B3LYP/6-31+G(d,p) level, then cal. δ_C was scaling corrected from the calculated shielding tensor (σ) using the formula $\delta_C = (186.2534-\sigma)/1.0496$.

Table S2. Inhibition rate of **1** on *Eleusine indica* seed germination

	Inhibition rate (%)	test concentration (μg/mL)						
		10	20	40	60	80	100	IC_{50} (μg/mL)
Compound **1**	radicle length	-1.42 ± 10.37	-4.64 ± 11.50	5.35 ± 11.08	15.84 ± 5.04	25.23 ± 19.30	29.19 ± 5.94	97.72
		20	40	60	80	100	200	IC_{50} (μg/mL)
Glyphosate	germ length	32.40 ± 7.75	48.12 ± 8.51	56.18 ± 4.56	60.66 ± 4.86	59.30 ± 7.84	71.26 ± 10.59	44.67
	radicle length	30.14 ± 9.33	56.31 ± 9.34	62.62 ± 5.16	63.96 ± 8.27	60.38 ± 3.00	75.09 ± 7.94	38.90

Data were presented as mean ± STD

Table S3. Inhibition rate of **2** on *Amaranthus retroflexus* L. seed germination

	Inhibition rate (%)	test concentration (μg/mL)						
		10	20	40	60	80	100	IC_{50} (μg/mL)
Compound 2	germ length	-19.64 ± 11.88	-12.71 ± 22.42	11.95 ± 7.26	21.73 ± 6.98	38.67 ± 5.28	78.11 ± 6.48	79.43
	radicle length	5.00 ± 13.16	24.54 ± 18.14	48.75 ± 7.30	64.23 ± 12.74	69.44 ± 7.24	78.36 ± 10.40	43.65
		20	40	60	80	100	200	IC_{50} (μg/mL)
Glyphosate	germ length	42.69 ± 9.10	55.94 ± 7.46	58.52 ± 3.87	62.56 ± 3.92	68.27 ± 5.48	72.30 ± 4.68	30.55
	radicle length	10.47 ± 12.34	27.55 ± 5.32	43.90 ± 6.96	50.41 ± 10.69	60.16 ± 1.38	80.04 ± 7.61	75.96

Data were presented as mean ± STD
